# Characteristics of infants born to mothers with autoimmune disorders

**DOI:** 10.1186/1546-0096-12-S1-P115

**Published:** 2014-09-17

**Authors:** Takayuki Kishi, Takako Miyamae, Satoru Nagata, Hisashi Yamanaka

**Affiliations:** 1 Department of Pediatrics, Tokyo Women's Medical University, Tokyo, Japan; 2 Institute of Rheumatology, Tokyo Women's Medical University, Tokyo, Japan

## Introduction

Autoimmune diseases frequently affect women of childbearing age. Gravidas with autoimmune diseases have a tendency to develop complications, such as pregnancy-induced hypertension in pregnancies complicated by systemic lupus erythematosus (SLE). The activity of autoimmune diseases is often exacerbated during pregnancy. The transplacental passage of autoantibodies may have an effect on the fetus or neonate; congenital heart block, in particular, is often irreversible. Therefore, increased vigilance is required for pregnancies complicated by autoimmune disorders.

## Objectives

Herein the characteristics of newborns from mothers with autoimmune disorders were evaluated.

## Methods

Clinical manifestations were assessed in 40 newborn infants from 38 mothers with autoimmune disororders (20 with SLE, 10 with rheumatoid arthritis, 6 with anti-phospholipid syndrome, 6 with Sjogren’s syndrome, 4 with screloderma, 1 with mixed connective tissue disease, and 1 with juvenile dermatomyositis) who attended the Tokyo Women’s Medical University Hospital between January 2011 and November 2013. The following information deriverd from the patients’ charts was reviewed: maternal autoimmune disease, maternal autoantibodies, medications prescribed during pregnancy, gestational age (GA), and autoantibodies transferred to the infant.

## Results

The average age of the mother at the time of the delivery was 33.7 years. Twenty mothers had anti-Ro/SS-A, 5 had anti-La/SS-B, and 4 had anti-RNP antibodies. Twenty-two gravidas (57.9%) were treated with oral glucocorticoids (GC) during pregnancy. The average dose of predonisolone and methylprednisolone was 10.8 mg/day (2-30 mg/day) and 8.0 mg/day (4-16 mg/day), respectively. Four gravidas with SLE used immunosuppresants (cyclosporine[n=2], tacrolimus [n=2]). Seven of 38 gravidas (18.7%) were delivered by emergency cesarean section. The average GA at birth was 36 3/7 weeks (23 6/7-41 1/7 weeks) and 17 of 40 (42.5%) had preterm births. Four of 23 term newborns met the criteria for small for gestational age (SGA). The average birth weight was 2314±649 g (range, 508-3676 g), and included 23 low birth weight (LBW) newborns (57.5%) and 3 extremely LBW newborns (7.9%). When the gravida used GC during pregnancy, there were significantly higher rates of preterm births and SGA neonates (p<0.05). The birth weights and GAs were not significantly different between gravidas with and without SLE. The average GA and birth weight of newborns whose mothers were treated with GC was 35 5/7 weeks and 2131±704 g, respectively, suggesting that GC may be a factor that affects GA and birth weight. Autoantibodies were detected in 18 infants (anti-Ro/SS-A [18/20; 90%] and anti-La/SS-B [3/5; 80%]). One baby had congenital complete heart block. Rash, thrombocytopenia, and hyper-transaminasemia (ALT > 35 U/l) developed after hospital discharge in 1, 3, and 4 infants, respectively. With respect to neonates born to antiSS-A antibody-positive mothers, maternal GC use or non-use had no effect on the incidence of autoantibodies and infant symptoms. There were no infants who developed congenital heart block born to mothers who had used prednisolone during pregnancy.

## Conclusion

The probabilities of preterm delivery and LBW infants were higher and more remarkable in gravidas treated with GC. Further research is needed to better understand how maternal autoimmune disease activity influences the fetus and neonate. Continued observation by pediatricians is important until autoantibodies are cleared in the infant.

## Disclosure of interest

None declared.

